# Social Intelligence and Psychological Distress: Subjective and Psychological Well-Being as Mediators

**DOI:** 10.3390/ijerph17217785

**Published:** 2020-10-24

**Authors:** Carolina M. Azañedo, Santiago Sastre, Teresa Artola, Jesús M. Alvarado, Amelia Jiménez-Blanco

**Affiliations:** 1 Department of Psychology, Villanueva University, 28034 Madrid, Spain; cmartin@villanueva.edu (C.M.A.); ssastre@villanueva.edu (S.S.); tartola@villanueva.edu (T.A.); 2 School of Psychology, Complutense University of Madrid, 28223 Madrid, Spain; ajimblangr@ucm.es

**Keywords:** social intelligence, subjective well-being, psychological well-being, psychological distress

## Abstract

The strength named “social intelligence” in the Values in Action (VIA) Classification of Character Strengths and Virtues represents emotional, personal, and social intelligences, which are considered “hot intelligences”. This work contributed to the study of the mechanisms of influence of social intelligence on mental health. A multiple mediation model was proposed to quantify the direct effect of social intelligence on psychopathological symptoms, as well as its indirect effect through its impact on components of subjective and psychological well-being. This study involved 1407 university students who completed the Values in Action Inventory of Strengths (VIA-IS), the Satisfaction with Life Scale (SWLS), the Positive and Negative Affect Schedule (PANAS), the Psychological Well-Being Scales (PWBS), and the Symptom Checklist-90-Revised (SCL-90-R). Social intelligence was found to be significantly associated with life satisfaction (*a* = 0.33, *p* < 0.001), positive affect (*a* = 0.42, *p* < 0.001), and negative affect (*a* = −0.21, *p* < 0.001), transmitting significant indirect effects on psychopathological symptomatology through these components of subjective well-being. Likewise, social intelligence was positively and significantly related to psychological well-being (*a*-paths ranged from 0.31 to 0.43, *p* < 0.001), exerting significant and negative indirect effects on psychological distress through the dimension of positive relations with other people. These results could be useful in order to expand the explanatory models of the influence of social intelligence on mental health and to design interventions based on this strength for the promotion of well-being and the reduction in psychological distress.

## 1. Introduction

Emotional, personal, and social intelligences fit the description of hot, broad intelligences. They have in common “their concern for the human world of inner experience and outer relationships” [1] (p. 295). All three constitute one of the strengths included in the Values in Action (VIA) Classification of Character Strengths and Virtues [2], which was named “Social Intelligence” (SI) to emphasize its relevance at the interpersonal level.

Previous research has shown that character strengths have strong connections with well-being in adults (e.g., [3,4,5,6,7]), youths, and children (e.g., [8,9,10]). Two forms of well-being have been distinguished: subjective well-being (SWB) and psychological well-being (PWB) [11,12]. The SWB perspective defines well-being in terms of life satisfaction and balance between positive affect and negative affect. Life satisfaction, the cognitive aspect of SWB involves global judgments of one’s life; positive and negative affect, the affective components of SWB, refer to experiencing pleasant or unpleasant emotions, respectively [13]. Regarding PWB, Ryff [14] proposed a multidimensional model that included six distinct dimensions of positive psychological functioning: self-acceptance, positive relations with others, autonomy, environmental mastery, purpose in life, and personal growth.

Most studies on the relationship between character strengths and well-being have focused on SWB components and less on PWB dimensions as indicators of well-being [15]. Probably, the well-being variable with which strengths have been most frequently related has been life satisfaction. Numerous studies have found a significant (positive) correlation between this cognitive component of SWB and SI (e.g., [7,16,17,18]). In addition, most of the evidence so far reflects that SI is related to the affective components of SWB, with the absolute values corresponding to the correlations with positive affect being greater than those with negative affect (e.g., [16,17,19]). With respect to PWB, the dimensions of environmental mastery, purpose in life, and self-acceptance showed the highest positive associations with character strengths [20]. Hausler et al. [15] found a moderate positive correlation between SI and the PWB total score; this character strength showed the strongest relationship with the dimension of environmental mastery.

The interaction between well-being and psychopathology has received considerable attention. Both are indicators of overall mental health. Ryff [21] conducted a research review around the connections between well-being and health that revealed the role of PWB dimensions as protective of mental problems (e.g., [22,23,24,25]). Some of these studies revealed that a variety of mental problems and disorders (such as depression, anxiety disorder, schizophrenia, or post-traumatic stress disorder) were associated with lower levels of PWB (e.g., [26,27,28,29]).

Regarding SWB, one of the most relevant contributions to research on the affective component of well-being was the broaden-and-build theory of positive emotions [30]. This theory proposes that experiencing positive emotions broadens the repertoires of thought and action; this broadening favors the construction of personal resources to face situations, making the person more resistant to difficulties, more creative, and strengthening his or her social integration; this, in turn, leads to experiencing new positive emotions, thus generating a growing spiral. Therefore, positive emotions help to increase the resources related to the well-being, happiness, and success of people [31]. Positive affect promotes optimal psychological functioning, enhancing cognitive and emotional resources, fostering interpersonal relationships [31,32,33,34], and generating better health [35]. Interventions aimed at promoting positive emotions help prevent and treat problems related to depression, anxiety, aggression, and stress [36].

The fact that strengths use enhances well-being and that different well-being process protect against psychological problems could imply that well-being may mediate the link between SI and mental health. The purpose of the present study was a more fine-grained analysis examining the relationship between SI and well-being in affecting psychological distress. Specifically, our aim was to examine the direct and indirect effects of SI on psychological distress through the components and dimensions of SWB and PWB. We proposed a parallel multiple mediator model in which SI was modeled as influencing psychological distress indirectly through nine mediators: the cognitive and affective components of SWB (i.e., life satisfaction, positive affect, and negative affect) and the six dimensions of PWB (i.e., self-acceptance, positive relations with others, autonomy, environmental mastery, purpose in life, and personal growth). To our knowledge, this research has not been carried out before. We hypothesized that SWB and PWB mediate the relationship between SI and psychological distress.

## 2. Method

### 2.1. Participants

The sample consisted of 1407 Spanish-speaking students (781 women, 626 men) from different places in Spain. Specifically, participants were recruited from five Spanish universities and their mean age was 34.36 years (*SD* = 10.80; range 18–74 years). The inclusion criteria to be part of the research sample were (a) being 18 years old or older, (b) being a university student enrolled in a degree in psychology, and (c) providing informed consent to take part in the study. Table 1 presents the quantifiable information for the sociodemographic characteristics.

### 2.2. Procedure

The participants completed a battery of questionnaires including measures of sociodemographic information (i.e., age, gender, and marital status), academic information (i.e., educational level), character strengths (specifically, SI), SWB and PWB, and symptoms of psychopathology. The data collection and the administration of the questionnaires were carried out online. Students were recruited through a study invitation email sent to their university email address. The recruitment message included a link to the study website, from which potential participants could access informed consent and online questionnaires. 

The present study protocol was authorized by the Ethics Committee of the Villanueva University (Madrid, Spain), with the reference 2020-27. All the potential study participants were provided with pertinent information they needed to know about the research to give a voluntary informed consent to participate in the study. This permission for inclusion was submitted online before they filled out the questionnaires. Students were advised that the task of completing all the questionnaires would take approximately one hour in total. This time could be freely distributed at as many intervals as deemed necessary. All the individuals were informed of their right to refuse to participate or to withdraw from the task of filling out the questionnaires without penalty and of whom to contact for information if they had any questions or problems. The consent form indicated that the study data (i.e., individual responses to the questionnaires) were anonymous. No identifiers (e.g., name, phone number, address) were collected that linked responses to a specific participant.

### 2.3. Instruments 

The VIA Inventory of Strengths (VIA-IS) [1,37] is a 240-item self-report which measures 24 character strengths, including SI. In the current study, we applied the Spanish adaptation of the VIA-IS [16]. Each item is rated on a five-point scale ranging from 1 (very much unlike me) to 5 (very much like me). In the present study, we only used the score obtained in the subscale of SI, which was calculated for each participant by averaging the ratings of the SI items. A higher score indicates a higher degree of endorsement of SI. The Cronbach alpha coefficient for this subscale was 0.77 in the current sample.

The Satisfaction with Life Scale (SWLS) [38] is a 5-item measure of the life satisfaction component of SWB. In the present study, we used the Spanish version of the SWLS [39]. Individuals rated each item on a five-point scale ranging from 1 (totally disagree) to 5 (totally agree). One final score was calculated for each participant by averaging the ratings of the five items. A higher score indicates a higher degree of satisfaction with life in general. In the present sample, the scale’s reliability was satisfactory (*α* = 0.88).

The Positive and Negative Affect Schedule (PANAS) [40] is a 20-item measure of the affective component of SWB. The PANAS consists of a Positive Affect (PA) subscale with ten adjectives representing positive affect, and a Negative Affect (NA) subscale with other adjectives indicating negative affect. This questionnaire uses a five-point Likert scale, ranging from 1 (very slightly or not at all) to 5 (extremely). Subscale scores were averaged across items, with a higher score indicating higher degree of the specific affect dimension. In the current sample, the Cronbach alpha coefficient was 0.92 for the PA subscale and 0.89 for the NA subscale.

The Psychological Well-Being Scales (PWBS) [41] is a self-report instrument which has six scales: self-acceptance, positive relations with others, autonomy, environmental mastery, purpose in life, and personal growth. In this study, we used the shortened version proposed by Díaz et al. [42]. The six scales were 29 items in total, and the response range was from 1 (totally disagree) to 6 (totally agree). In the current sample, the internal consistency (*α*) coefficients for each scale were 0.84 for self-acceptance and purpose in life, 0.80 for positive relations with others, 0.74 for autonomy and personal growth, and 0.73 for environmental mastery. A high score reflects a high sense of the specific PWB dimension.

The Symptom Checklist-90-Revised (SCL-90-R) [43] is a 90-item self-report instrument which evaluates a broad range of psychological problems and symptoms of psychopathology. This questionnaire measures the dimensions of somatization, obsessive-compulsive symptoms, interpersonal sensitivity, depression, anxiety, hostility, phobic anxiety, paranoid ideation, and psychoticism. Each item is rated on a five-point scale ranging from 0 (not at all) to 4 (extremely). In this study, we used the Spanish adaptation of the SCL-90-R [44]. In the current sample, all the dimensions showed good levels of internal consistency (with Cronbach’s *α* ranging from 0.76 to 0.91). One of the summary scores that this instrument provides is the Global Severity Index (GSI), which is obtained by summing the scores on the nine dimensions and dividing this score by the total number of items. We used the GSI to measure overall psychological distress. This global index is the best single indicator of the severity of a disorder and should be used in most instances where a single summary score is needed [43].

We selected these questionnaires and used them for data collection because they are standardized and validated instruments that provide an efficient way of obtaining the necessary information about the study variables.

### 2.4. Data Analysis

The SPSS software (version 25) was used for the statistical analysis. An exploratory data analysis was carried out and the normal distribution of the study variables was confirmed. As part of the process of preparing data for analysis, we performed data cleaning to detect possible cases in the database that did not meet the inclusion criteria, missing data, and outliers. No such cases or data were found. Descriptive statistics (mean scores and standard deviations) of the study variables were calculated. Pearson’s correlation coefficient was used to determine the association between SI, components of SWB, dimensions of PWB, and the index GSI. The PROCESS tool [45] (version 19) in SPSS was applied to conduct multiple mediation analysis to determine if the effect of SI on GSI was mediated through SWB and PWB.

## 3. Results

### 3.1. Descriptive Statistics and Correlations between Study Variables 

Table 2 presents the descriptive statistics (mean and standard deviation) and correlations between SI, components of SWB, dimensions of PWB, and psychological distress. SI had significant correlations with every component or dimension of well-being. These correlations ranged from −0.20 (negative affect) through to 0.43 (self-acceptance). SI was significantly and negatively associated with psychological distress (*r* = −0.22). Regarding the correlations between well-being variables, except for negative affect, all the coefficients were positive and greater than 0.25. The highest correlation was found between self-acceptance and purpose in life (*r* = 0.79). The correlation between psychological distress and negative affect was positive and strong (*r* = 0.72). In contrast, the correlations between psychological distress and the other well-being indicators were negative, ranging from −0.29 (personal growth) to −0.56 (self-acceptance and environmental mastery). These correlation patterns were not affected by gender and education level.

### 3.2. Multiple Mediation Analysis

A multiple mediation analysis was conducted using Model 4 in the version 3.5 of the PROCESS tool [45] in SPSS to determine whether the effect of SI on psychological distress was mediated through the well-being variables. We controlled for gender and age (as covariates) in the analysis. A parallel multiple mediator model was tested in which SI was the independent variable (X), the components of SWB (i.e., satisfaction with life, positive affect, and negative affect) and the dimensions of PWB (i.e., self-acceptance, positive relations with others, autonomy, environmental mastery, purpose in life, and personal growth) were the potential mediators (M), and psychological distress was the dependent variable (Y). The inclusion of these mediators simultaneously in an integrated model allowed for a formal comparison of the size of the indirect effects of SI through them. Bias-corrected (BC) bootstrapping (with 10,000 resamples) was used to generate confidence intervals (CIs) for the hypothesis tested. A conservative significance level of 0.001 was adopted in this analysis. 

Standardized regression coefficients were calculated for each path in this mediation model. Path *a* estimated the effect of X on each mediator M, path *b* estimated the effect of each M on Y controlling for X, path *c* estimated the total effect of X on Y, and path *c’* estimated the effect of X on Y holding all the M variables constant. A multiple mediation strategy allowed the estimation of both the total indirect effect associated with all mediators and the specific indirect effect associated with each mediator. Specific indirect effects were calculated as the product of the regression coefficients corresponding to paths *a* and *b*. When zero was not contained in the bias-corrected 99% CI, it was concluded that the indirect effect of X on Y through M was significant. Pairwise contrasts of all the specific indirect effects involved in the model were also calculated. 

**Figure 1:**
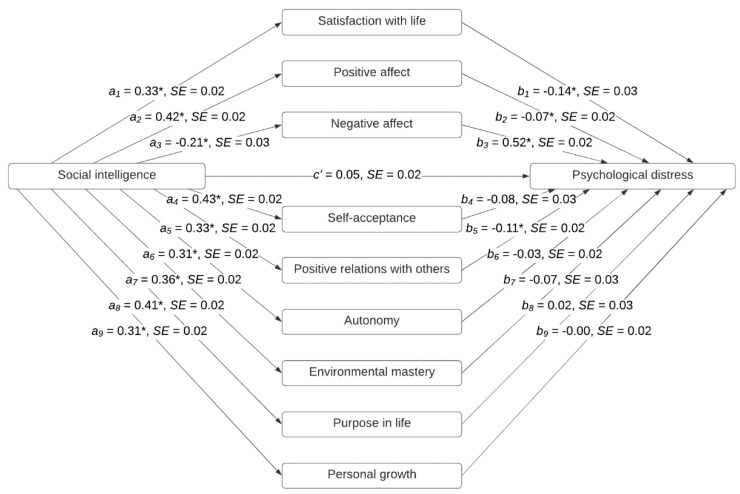
Parallel multiple mediator model of social intelligence, subjective well-being, psychological well-being, and psychological distress. Standardized coefficients and standard errors (*SE*) are shown in the figure. * *p* < 0.001.

Figure 1 shows the parallel multiple mediator model. The *a*-paths were all significant and ranged from −0.21 to 0.43. The *b*-paths were only significant for satisfaction with life, positive affect, negative affect, and positive relations with others. The total effect was significant, but the direct effect of SI on GSI was not. The BC 99% CI of the specific indirect effect of satisfaction with life (*ab* = −0.05, BC 99% CI = −0.08 to −0.02), positive affect (*ab* = −0.03, BC 99% CI = −0.06 to −0.01), negative affect (*ab* = −0.11, BC 99% CI = −0.15 to −0.07), and positive relations with others (*ab* = −0.04, BC 99% CI = −0.06 to −0.02) did not contain zero. These results indicated that satisfaction with life, positive affect, negative affect, and positive relations with others mediated the relationship between SI and psychological distress. In contrast, self-acceptance, autonomy, environmental mastery, purpose in life, and personal growth did not emerge as significant mediators between SI and GSI. The BC 99% CIs of the pairwise contrasts between the specific indirect effects revealed that negative affect was a stronger mediator than positive affect (*ab* = −0.08, BC 99% CI = 0.03 to 0.13), satisfaction with life (*ab* = −0.06, BC 99% CI = 0.02 to 0.11), and positive relations (*ab* = −0.07, BC 99% CI = −0.11 to −0.03). Pairwise contrasts also revealed that there were no significant differences in the magnitude of the mediating effects of satisfaction with life, positive affect, and positive relations with others. The same parallel multiple mediator model was tested for women and men separately (see Appendix A). No major gender differences were found in the results of these mediation analyses.

## 4. Discussion

The aim of the present study was to examine whether SI was effective in enhancing well-being indicators and whether these processes mediated the effects on GSI. Our hypothesis was partly supported. The results indicated that SI was positively linked with SWB and PWB, which was in line with earlier studies demonstrating the relationship between character strengths and well-being (e.g., [6,15,18]). Regarding the relationship between well-being and psychopathological symptoms, the results of the mediation analysis showed that the *b*-paths were significant for every component of SWB (i.e., life satisfaction, positive affect, and negative affect), but only for one of the six dimensions of PWB (i.e., positive relations with others). When the nine dimensions of well-being were included simultaneously in the parallel multiple mediator model, the effect of SI on psychological distress was uniquely mediated through SWB and positive relations with others. The mediating role of negative affect was mainly stronger compared to the mediating role of positive affect, satisfaction with life, and positive relations. In addition, the mediation analysis suggested that SI did not influence psychopathological symptoms directly, but only indirectly through the above-mentioned indicators. The patterns of correlation between study variables in the different subsamples (i.e., according to gender and educational level) were similar, and no differences were observed between men and women in the proposed mediation model.

A growing body of research found that mental well-being may protect against the onset of psychopathological symptoms (e.g., [21,24,46,47]). Therefore, a possible strategy for the prevention of psychological distress is to promote mental well-being processes [24,48]. The present study corroborates evidence about the strong positive relationship between affect balance and mental health. The prominent role of positive emotions in mental health has been underscored by previous research and theoretical frameworks, such as broaden-and-build theory [30] mentioned above. Social support and positive relations with others have been associated with lower degrees of psychopathological symptoms (e.g., [49,50]), and they are thought to protect against psychological distress [51,52]. Taken together, affect balance and positive relations seem to be of utmost importance in mental health, suggesting that it is fruitful to strengthen them. This study reveals that one potential way to achieve that goal could be by fostering SI through targeted interventions. In line with this, the approach of positive psychotherapy (PPT) [53] offers an effective way to prevent and treat psychopathological symptoms. According to PPT, building character strengths such as SI and positive emotions may counterbalance psychological distress. Our results are congruent with what is proposed by this approach. Social and emotional intelligence is a resource that has positive consequences for personal and social well-being [54,55]. The ability to recognize feelings in oneself and in others contributes to managing successful social interactions with other people, promoting positive emotions and improving the global cognitive evaluation of one’s life satisfaction [56]. In turn, affect balance and positive relations with others are mechanisms underlying mental health. Designing positive interventions to cultivate these personal and social resources will result in less psychopathological symptoms, greater well-being and better overall state of mental health.

This research provides empirical evidence with regard to the beneficial effects of SI in promoting mental health, enhancing specific well-being process, and preventing or mitigating psychological problems. Nevertheless, caution should be kept in the interpretation of these findings because of some limitations of the current study. First, the measures used in this study were based solely on self-report questions, where response bias (e.g., social desirability bias, tendency to respond randomly to items) could be of special concern. We attempted to minimize the risk of response bias by proposing the task of answering the questionnaires as entirely voluntary and by informing participants beforehand that the data collection would be anonymous. Second, the cross-sectional design we used in this research prevented the unequivocal establishment of a causal relationship. The present study involved data collection from a single sample at one specific point in time, so in this case “theory or solid argument is the only foundation upon which a causal claim can be built” [45] (p. 81). Consequently, it will be necessary in future studies for mediation analyses to be performed within the context of longitudinal designs. Third, one of the strengths of our study was the large sample size. However, the participants were university students, so future research needs to investigate whether the proposed mediation model can be generalized to other populations. Fourth, we cannot exclude the possibility that other variables could be affecting the nine well-being processes as well as psychological distress. Therefore, a next direction for research could be to include other possible mediators in the model.

## 5. Conclusions

This cross-sectional investigation contributed to elucidate the relationship between SI and mental health. This study added to previous work by using mediation modeling to investigate the possible internal mechanisms of SI in affecting psychological distress. The results from a multiple mediation analysis demonstrated that not all well-being processes emerged as mechanisms of change for establishing improvements in psychopathological symptoms. Evidence of significant indirect effects was present only throughout four of the nine well-being indicators: satisfaction with life, positive affect, negative affect, and positive relations with others. Specifically, the strength of SI contributes to the improvement of affect balance (i.e., experiencing more pleasant emotions than unpleasant ones), global judgments of one’s life, and quality relations with others, which in turn resulted in less psychological distress. However, independent of these mechanisms, there was no evidence that SI influenced psychopathological symptoms by changing self-acceptance, autonomy, environmental mastery, purpose in life, and personal growth.

To conclude, we can say that these findings endorsed the role of SI as a contributor to mental health. Our study provides further support for the importance of enhancing and improving individuals’ SI as a strategy to promote optimal mental well-being and prevent mental disorders. In particular, SI may reduce psychological distress by increasing mainly SWB. These results could be useful to develop and expand explanatory models of the impact of social intelligence on well-being and psychopathological symptomatology. Additionally, knowledge of this evidence could help to design and develop effective interventions for improving mental health in a variety of settings, not only in clinical work, based on the promotion of SI. The application of positive interventions to cultivate SI could lead us to experience less psychological distress and to achieve a state of complete well-being.

## Figures and Tables

**Table 1:** Sociodemographic characteristics of the sample.

		Women		Men		Total	
		*Fr*	*%*	*Fr*	*%*	*Fr*	*%*
Age	18–24	229	29.32	111	17.73	340	24.16
	25–34	224	28.68	170	27.16	394	28.00
	35–44	206	26.38	191	30.51	397	28.22
	45–54	106	13.57	119	19.01	225	15.99
	55–74	16	2.05	35	5.59	51	3.62
		781	100	626	100	1407	100
Marital status	Single	402	51.47	306	48.88	708	50.32
	Married	207	26.50	197	31.47	404	28.71
	Living as a couple without being married	116	14.85	85	13.58	201	14.29
	Divorced or separated	53	6.79	36	5.75	89	6.33
	Widowed	3	0.38	2	0.32	5	0.36
		781	100	626	100	1407	100
Educational level	High-school education	311	39.82	240	38.34	551	39.16
	Postsecondary education	127	16.26	97	15.50	224	15.92
	University degree	336	43.02	276	44.09	612	43.50
	Ph.D. degree	7	0.90	13	2.08	20	1.42
		781	100	626	100	1407	100

Note. Fr = frequency or number of participants in each group. % = percentage of participants in each group.

**Table 2:** Descriptive statistics and Pearson correlations between social intelligence, components of subjective well-being, dimensions of psychological well-being, and psychological distress.

		Pearson Correlations									
Variables	1	2	3	4	5	6	7	8	9	10	11
1. Social intelligence	—										
2. Satisfaction with life	0.32 *	—									
3. Positive affect	0.41 *	0.52 *	—								
4. Negative affect	−0.20 *	−0.45 *	−0.30 *	—							
5. Self-acceptance	0.43 *	0.74 *	0.58 *	−0.47 *	—						
6. Positive relations	0.32 *	0.39 *	0.31 *	−0.27 *	0.46 *	—					
7. Autonomy	0.30 *	0.27 *	0.28 *	−0.34 *	0.44 *	0.26 *	—				
8. Environmental mastery	0.36 *	0.67 *	0.56 *	−0.47 *	0.76 *	0.47 *	0.42 *	—			
9. Purpose in life	0.41 *	0.65 *	0.56 *	−0.38 *	0.79 *	0.42 *	0.34 *	0.76 *	—		
10. Personal growth	0.30 *	0.35 *	0.38 *	−0.21 *	0.55 *	0.34 *	0.34 *	0.51 *	0.54 *	—	
11. Psychological distress	−0.22 *	−0.55 *	−0.43 *	0.72 *	−0.56 *	−0.36 *	−0.36 *	−0.56 *	−0.47 *	−0.29 *	—
*Mean*	3.80	3.57	3.49	1.95	4.63	4.63	4.19	4.49	4.61	5.12	0.58
*Standard Deviation*	0.54	0.85	0.78	0.76	0.97	1.02	0.89	0.89	0.93	0.81	0.51

* *p* < 0.001.

**Figure A1:**
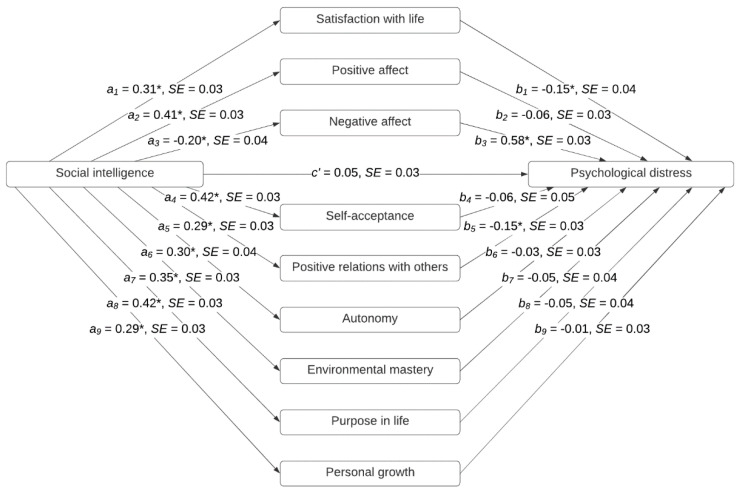
Parallel multiple mediator model of social intelligence, subjective well-being, psychological well-being, and psychological distress for women. Standardized coefficients and standard errors (*SE*) are shown in the figure. * *p* < 0.001.

**Figure A2:**
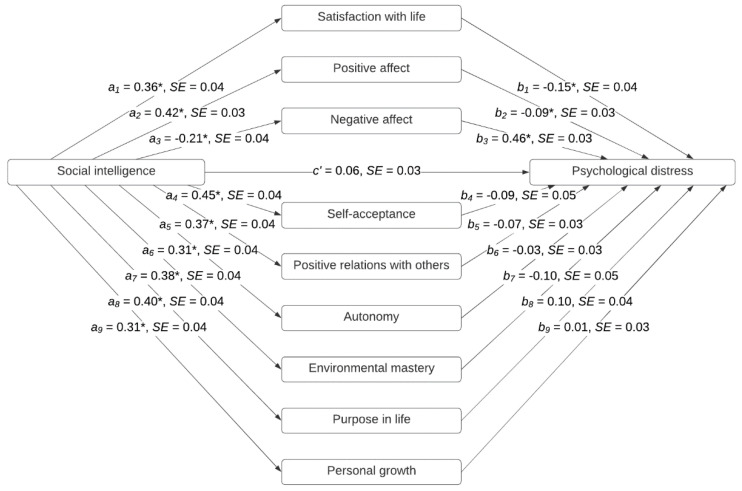
Parallel multiple mediator model of social intelligence, subjective well-being, psychological well-being, and psychological distress for men. Standardized coefficients and standard errors (*SE*) are shown in the figure. * *p* < 0.001.

## Appendix A

Figure A1 and Figure A2 show the parallel multiple mediator model for women and men, respectively. The values obtained for these mediation models did not reveal large differences in terms of gender.
